# Cost comparison of transcatheter and operative closures for patients with secundum atrial septal defects in Vietnam

**DOI:** 10.1016/j.heliyon.2021.e05904

**Published:** 2021-01-08

**Authors:** Ho Xuan Tuan, Phan The Phuoc Long, Vu Duy Kien, Harald Kramer, Robert Dalla-Pozza

**Affiliations:** a Center for International Health, Ludwig-Maximilians-University, Munich, Germany; b School of Medicine and Pharmacy, The Da Nang University, Da Nang city, Viet Nam; c OnCare Medical Technology Company Limited, Ha Noi, Viet Nam; d Institute of Clinical Radiology, University Hospital of Munich, Germany; e Department of Pediatric Cardiology and Pediatric Intensive Care Medicine, Großhadern Clinic, Ludwig Maximilians University, Munich, Germany

**Keywords:** Cost, Atrial septal defect, Secundum ASD, Operative closure, Transcatheter closure, Vietnam

## Abstract

We aim to estimate and compare the costs of operative and transcatheter closure for patients with secundum atrial septal defect (ASD) in Vietnam. This was a retrospective cross-sectional study based on medical records of congenital heart diseases (CHD) patients in Da Nang Hospital, Vietnam from 2010 through 2015. All costs in this study were calculated according to a provider's perspective. All pricing data were converted into USD at the 2015 exchange rate. A total of 258 patients with secundum ASD were recruited in the study, including 35 patients treated by operative closure and 223 patients treated by transcatheter closure. The total treatment costs of the transcatheter closure group (US $3,107.9) were higher than those of the operative closure group (US $2,080.5). The cost of the procedure and medical supplies accounted for 67.3% of the total treatment cost in the operative closure group, while the cost of occlusion devices accounted for 62.2% of the total cost in the transcatheter closure group. Given the advantages of the transcatheter closure procedure, reducing occlusion device costs may increase the proportion of patients treated with this technique.

## 1 Introduction

Atrial septal defect (ASD) is one of the most common heart defects among patients with congenital heart diseases (CHD) [1,2,3], and, of these patients, most have secundum ASD [4]. In the past, secundum ASD treatment was mainly by operative closure, while in past few decades, transcatheter closure has been developed to provide an additional treatment option [5]. Device closure of secundum ASD has gradually improved to increase efficiency, reduce the risk of adverse events and, in particular, reduce treatment costs [6,7]. Currently, both operative and transcatheter closures are typically offered. While transcatheter closure is less invasive than operative closure, secundum ASD patient anatomical requirements exclude some patients from transcatheter closure treatment [6,8]. Previous studies have suggested that transcatheter closure treatment required a shorter hospital stay, but it was as effective as operative closure [9,10]. While the choice between operative and transcatheter closure procedures depends on many factors, treatment costs may play an important role, especially in developing countries.

A previous study in Vietnam showed that ASD accounted for a substantial proportion (18.5%) of CHD patients [11], indicating a considerable number of ASD patients requiring hospital intervention. In Vietnam, while operative closure is still being used, transcatheter closure has become more common in treating secundum ASD in the past decade. Furthermore, while studies conducted in other countries compared the costs of operative and transcatheter closures for patients with secundum ASD [12,13,14,15], the results of these studies varied widely depending on study methods and countries. To date, no studies assessing the costs of operative and transcatheter closure procedures in patients with secundum ASD in Vietnam have been conducted. As such, this study aims to estimate and compare the costs of operative and transcatheter closure for patients with secundum ASD in Vietnam.

## 2 Methods

### 2.1 Study setting

The study was conducted in Da Nang Hospital (Da Nang, Vietnam), one of the largest central Vietnam hospitals. Da Nang Hospital is a general hospital with various specializations that include a department specializing in the treatment of all types of cardiovascular disease. Most patients with cardiovascular diseases in the south of central Vietnam visit Da Nang Hospital for diagnosis and treatment.

### 2.2 Data sources and data collection

This was a cross-sectional study based on the medical records of CHD patients in Da Nang hospital, that included six years of retrospective data (01/01/2010 through 31/12/2015). The study was approved by the Ethical Committee Board of Da Nang Hospital in Vietnam (No. 380/BVĐN-YĐ; dated 18/07/2016) and the Ludwig Maximilian University of Munich in Munich, Germany (No. 18–221; dated 02/05/2018). Inclusion criteria were CHD with secundum ASD diagnosis and treatment by either operative or transcatheter closure, and exclusion criteria were presence of additional congenital heart defects. Patient personal information was removed and kept confidential before analysis. General characteristics of patients were collected, including age, sex, and residence location. We examined patient medical records, operative or transcatheter closure reports, and collected information about defect size, length of hospital stay and complications following treatment.

### 2.3 Costs calculation

All costs in this study were calculated according to the perspective of the provider. Financial data were extracted from the Da Nang Hospital data with the assistance of the financial department. Hospital bills included line items such as hospital bed-days, laboratory tests, diagnostic imaging, therapeutics, medicines, supplies, and procedures. Quantity, unit cost, and total cost were described in detail on the analyzed medical bills. We grouped items into nine groups, including hospital bed-days, laboratory tests, diagnostic imaging, closure procedure, blood and blood products, medicine and fluids, medical supplies, occlusion devices, and treatment of complications. To estimate the cost of laboratory tests, diagnostic imaging, procedure, blood and blood products, medicine and fluids, medical supplies, and occlusion devices, we collected hospital bills for patients treated by operative and transcatheter closure. We categorized each item into the groups as mentioned earlier. The costs of hospital bed-days were estimated by using the average total days in hospital and the cost of each specific hospital bed type, including beds for before closure procedure, intensive care unit beds, and beds after the procedure. Costs are presented as means and minimum and maximum values. The cost of complication treatment was estimated by multiplying the probability of a specific complication event and the maximum cost to solve that event. We thus assumed an appropriate complication treatment costs for operative closure procedures at US $40 (range: $0–200) and transcatheter closure procedures at $29.7 (range: $0–1,500). There was no separate item for professional service in this study as the hospital utilized a billing package that included professional costs. Thus, professional costs were included in the procedure category. Since this study was retrospective, we only included direct treatment costs; all patients were discharged, so we could not collect relevant indirect costs (e.g., costs relating to transport or lost productivity of patients and their families).

### 2.4 Data analysis

Continuous variables were expressed here as means and standard deviations, and discrete variables were expressed as frequencies and percentages. Costing data were presented as means with minimum and maximum values. Student's t-tests and Chi-square tests were used to compare between operative and transcatheter closure group variables. All costs in this study were converted into 2015 USD (US $1 = VND 22,540). MS Excel (Microsoft Corporation, Redmond, WA, USA) and STATA version 14 (Stata Corp, College Station, TX, USA) were used for all data analyses. P-values < 0.05 were considered to indicate statistical significance.

## 3 Results

A total of 258 patients with secundum ASD were recruited to participate in the study, including 35 patients treated with operative closure and 223 patients treated with transcatheter closure. Table 1 shows the characteristics of patients with secundum ASD according to the closure procedures. There were no statistically significant differences according to sex, age, or residence between operative and transcatheter closure groups (all p > 0.05). However, the mean defect of the operative closure group was both longer and wider than that of the transcatheter group (both p < 0.01).

**Table 1:** Characteristics of patients with secundum atrial septal defects in this study.

	Operative (n = 35)	Transcatheter (n = 223)	p-value
	N (%) or mean (SD)		
Sex			
Women	22 (62.9)	159 (71.3)	0.31
Men	13 (37.1)	64 (28.7)	
Age (years)	22.4 (2.9)	20.1 (1.2)	0.49
Age group (years)			
<18	16 (45.7)	121 (54.3)	0.35
≥18	19 (54.3)	102 (45.7)	
Residence			
Urban	11 (31.4)	70 (31.3)	0.99
Rural	24 (68.6)	153 (68.7)	
Defect size (mm)			
Length	27.9 (8.1)	20.6 (7.6)	<0.01
Width	26.2 (7.8)	18.7 (7.5)	<0.01

SD: Standard Deviation.

Table 2 presents days in hospital and the occurrence of complications in the operative and transcatheter closure groups. The mean of total hospital days of the operative closure group (23.3 days) was statistically significantly more than that of the transcatheter closure group (15.4 days; p < 0.01). This difference was mainly related to hospital days after ASD closure, where mean hospital stay after the procedure was 3 days in the transcatheter closure group and 7.9 days in the operative closure group. In addition, patients in the operative closure group always required care in the intensive care unit (mean = 1.2 days). Complications were mainly septicemia (11.4%), heart failure (8.6%), and pneumonia (8.6%) in the operative closure group, and device embolization (0.9%) and transcatheter closure failure (2.2%) in the transcatheter closure group.

**Table 2:** Hospital days and complications in operative and transcatheter groups.

	Operative (n = 35)	Transcatheter (n = 223)	p-value
	N (%) or mean (SD)		
Hospital days			
Before procedure	14.2 (7.5)	12.3 (8.5)	0.20
Intensive care unit	1.2 (0.6)	NA	NA
After procedure	7.9 (3.5)	3.0 (3.6)	<0.01
Total hospital days	23.3 (9.1)	15.4 (8.9)	<0.01
Complication			
Pneumonia	3 (8.6)	0	NA
Septicemia (clinical symptom)	4 (11.4)	0	NA
Heart failure	3 (8.6)	0	NA
Incision infection	1 (2.9)	0	NA
Cerebrovascular accident	1 (2.9)	0	NA
Respiratory insufficiency	1 (2.9)	0	NA
Convulsion	1 (2.9)	0	NA
Device embolization	NA	2 (0.9)	NA
Transcatheter closure failure	NA	5 (2.2)	NA

NA: Not Applicable; SD: Standard Deviation.

**Figure 1:**
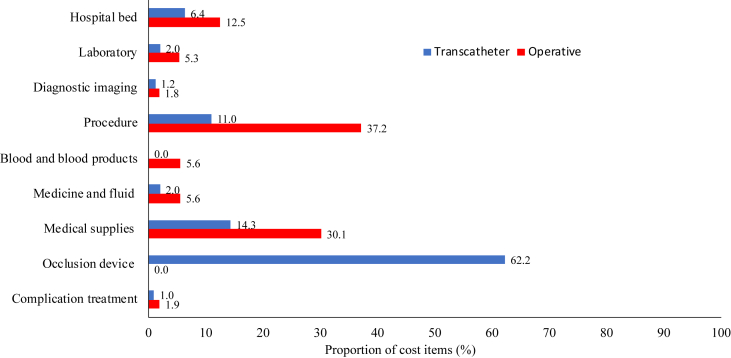
Proportion of costs by item according to closure procedure.

Table 3 shows operative or transcatheter closure treatment costs for patients with secundum ASD. The total cost in the transcatheter closure group (US $3,107.8) was higher than that of the operative closure group ($2080.5). For patients treated by operative closure, the most costly expenses were the procedure ($773.1) and medical supplies ($627.0). For a patient treated by transcatheter closure, the most costly expenses were the occlusion device ($1,934.3) and medical supplies ($445.3). If excluding the occlusion device costs, the total cost of the transcatheter closure procedure was $ 1,173.6, which was about 56.4% of the operative closure cost. The cost of the procedure and medical supplies accounted for 67.3% of the total cost in the operative closure group, while the cost of the occlusion device alone accounted for 62.2% of the total cost in the transcatheter closure group (Figure 1).

**Table 3:** Costs of treating patients with secundum atrial septal defects using operative or transcatheter closure procedures (2015 USD).

	Operative	Transcatheter
	Mean (Min-Max)	
Hospital bed	260.2 (77.6–413.4)	197.4 (9.0–569.1)
Laboratory	110.3 (97.0–126.7)	62.6 (43.8–91.5)
Diagnostic imaging	38.2 (31.3–41.7)	35.9 (33.0–37.3)
Procedure	773.1 (757.7–793.8)	341.0 (336.7–345.5)
Blood and blood products	116.2 (92.5–154.6)	NA
Medicine and fluid	115.5 (61.5–184.7)	61.7 (40.4–102.2)
Medical supplies	627.0 (616.4–644.2)	445.3 (352.4–492.0)
Occlusion device	NA	1,934.3
Treatment complications	40.0 (0.0–200.0)	29.7 (0.0–1500.0)
Total cost	2,080.5 (1734–2559.1)	3,107.8 (2,749.7–5,071.9)

NA: Not applicable.

## 4 Discussion

This is the first study to assess and compare the costs of operative and transcatheter closure procedures for patients with secundum ASD in Vietnam. Although costs were estimated according to hospital bills, they primarily reflected the cost for patients with secundum ASD being treated in hospital. The costs of transcatheter closure procedures were higher than those of operative closure procedures; however, this increase was mostly due to occlusion device costs. Determining the total costs and cost per item in the treatment of secundum ASD may help hospitals develop appropriate plans to treat patients with secundum ASD.

Although transcatheter closure procedures are considered a minimally invasive method and reduce the length of hospital stay for patients, the cost of this approach was higher than that of the operative closure procedure, something suggested by some previous studies [15,16,17], but not others [12,13,14,18,19]. The difference in costs between operative and transcatheter closure procedures, and the variation in cost-comparison study outcomes, might be attributed to previous studies conducted at different centers, and non-uniform inclusion of different items in the calculation of costs. In addition, the costs of occlusion devices also substantially affect the total costs of transcatheter closure procedure. In developing countries, occlusion devices must be imported from developed countries, raising the relative costs of these devices along with transcatheter closure treatment costs. In our study, if occlusion device costs were not taken into account, transcatheter closure procedure costs were only half that of operative closure costs. Thus, reduction of occlusion device costs could play an important role in reducing the total costs of treatment using transcatheter closure procedures for patients with secundum ASD.

These costs may also be affected by the timing of data collection, although all data were converted into USD, which may be considered a relatively stable benchmark currency. However, costs for operative and transcatheter closure procedures in lower- and middle-income countries were generally much lower than those in upper-income countries. While the cost of treating patients with secundum ASD in a lower-income country ranged from US $3,300-4,500 [17], the cost in the USA ranged from $7,000–15,000 [13] and, according to O'Byrne et al., up to $55,000–60,000 [12]. In upper- and middle-income countries, operative and transcatheter closure procedures costs ranged from $3,800-6,600 in Turkey and $8,500-12,000 in Brazil [14,15]. These differences could be explained by differences in costs assessment and regulation across countries and regulatory schemes. In Vietnam, the treatment costs for patients with secundum ASD were lower than those in middle- and upper-income countries, which could be explained by costs in Vietnam that are lower than in those countries, especially with regards to costs related to hospital bed-days and professional charges. Therefore, the cost of intervention for a patient with secundum ASD on average ranged from only $2,080-3,100 in Vietnam. Although the average cost of intervention for patients with secundum ASD was low in our study compared to other studies, this cost corresponds to Vietnam's yearly per capita GDP ($ 2,085.1 in 2015) [20]. Even so, treatment costs would be a financial burden for the majority of Vietnamese people. We believe that it is thus necessary to conduct an additional study to assess the cost-effectiveness of operative and transcatheter closure procedures in patients with secundum ASD, which may help hospitals develop improved treatment options for patients.

Currently, social health insurance in Vietnam has been implemented relatively well with a coverage rate of over 80% of the population [21,22]. Notwithstanding the high cost of treatment for patients with secundum ASD, a typical patient's actual payment was from 80 to 100% of the total cost for those patients who participated in social health insurance [21]. Some patients, who bypassed to a higher level according to the social health insurance system, had to suffer higher costs. In addition, if the relevant supplies and equipment were not covered by the insurance agency, the patient would still be liable for treatment costs. Due to the time and complexity of data collection required in this study, we did not explore the impact of social health insurance on the costs of operative and transcatheter closure procedures for patients with secundum ASD, and further studies would be needed to understand the impact of social health insurance in terms of financial protection for the treatment of patients with secundum ASD.

Complication also affected the costs of intervention for patients with secundum ASD. In our study, surgical-related complications were only reported with operative closure, which has previously been reported [23,24], which may increase the cost of treatment for patients. Although there were no surgery-related complications in the transcatheter closure procedure group, that group suffered complications relating to occlusion devices, including the device embolization and closure failure [25,26]. In these cases, patients must also undergo operative closure, thereby increasing the treatment costs. Moreover, in the long-term, erosion of device-related issues may also arise [27,28], so the cost of regular monitoring for patients treated with transcatheter closure may be noteworthy. Similarly, costs may also increase if occlusion devices require replacement after some period of time. In this study, we relied on average costs and the probability of complications for estimating the overall cost of treating a patient with secundum ASD. However, this may be an underestimate and might not reflect patients’ actual costs. Further studies should be conducted to understand better the various complications associated with different interventions in patients with secundum ASD.

We also report similarities with previous studies regarding study participant characteristics [12,13,14,15]. Specifically, study participants were more often female than male, relatively young, and more often from rural areas than from urban areas. The distribution of these factors within operative or transcatheter closure groups was relatively similar and differences were not statistically significant (all p > 0.05). However, the mean defect size in both dimensions of patients in the operative group was larger than that of patients in the transcatheter closure group. We recognize that this difference was common when conducting this type of study.

Our study also had some limitations. This study was performed in a single center with uniform practice, so care should be taken when applying our findings to other medical centers. Cost analyses were limited to direct medical costs according to hospital bills, so the total costs may thus be underestimated, given that patients may incur additional costs (e.g., drugs or other medical services). We did not measure patient's indirect costs (e.g., transport, lost productivity, or administration), so treatment under different contexts may affect costs of operative and transcatheter closure procedures in the short-term and long-term. The difference in defect size could also influence the indication between the two procedures. Therefore, this may also affect the results of the cost analysis in our study. In addition, despite the follow-up lasting many years, the sample size of our study was relatively small, thus limiting the generalization of the results.

## 5 Conclusions

We found that the cost of the transcatheter closure procedure was higher than that of the operative closure procedure in patients with secundum ASD. However, the majority of the cost of the transcatheter closure procedure was accounted for the occlusion device. Given the advantages of the transcatheter closure procedure, reducing the occlusion device's cost can increase the patients' likelihood of being treated with the transcatheter closure procedure. However, there is a need for studies with larger sample sizes and a more extensive follow-up after an intervention to better compare the overall costs of operative and transcatheter closure procedures.

## Declarations

### Author contribution statement

H. Tuan: Conceived and designed the experiments; Performed the experiments; Analyzed and interpreted the data; Contributed reagents, materials, analysis tools or data; Wrote the paper.

P. Long, H. Karmer and R. Dalla-Pozza: Conceived and designed the experiments; Analyzed and interpreted the data; Wrote the paper.

V. Kien: Conceived and designed the experiments; Analyzed and interpreted the data; Contributed reagents, materials, analysis tools or data; Wrote the paper.

### Funding statement

This research did not receive any specific grant from funding agencies in the public, commercial, or not-for-profit sectors.

### Data availability statement

Data will be made available on request.

### Declaration of interests statement

The authors declare no conflict of interest.

### Additional information

No additional information is available for this paper.
